# MiR-423-5p may regulate ovarian response to ovulation induction via CSF1

**DOI:** 10.1186/s12958-020-00585-0

**Published:** 2020-04-07

**Authors:** Shi Xie, Qiong Zhang, Jing Zhao, Jie Hao, Jing Fu, Yanping Li

**Affiliations:** 1 Reproductive Medicine Center, Xiangya Hospital, Central South University, 87 Xiangya Road, Changsha, Hunan China; 2 Clinical Research Center For Women’s Reproductive Health In Human Province, Changsha, Hunan China

**Keywords:** miR-423-5p, CSF1, Follicular development, In vitro fertilization (IVF)

## Abstract

**Background:**

We have previously shown that hsa-miR-423-5p expression in ovarian granulosa cells is decreased in high ovarian response populations. The objective of the present study was to find the target gene and mechanism for miR-423-5p involved in ovarian response regulation.

**Methods:**

(a) TargetScan was used to predict the target gene of hsa-miR-423-5p. (b) A model for hsa-miR-423-5p overexpression or inhibition was constructed by transfecting KGN cells with lentivirus. CSF1 mRNA and protein expression and luciferase activity were measured. (c) The cell cycles of control and lentivirus treated KGN cells were analyzed. Western blot was used to measure the expression of CDKN1A in KGN cells. (d) The concentration of E_2_ in KGN cell culture medium were measured.

**Results:**

(a) TargetScan revealed that the 3′ un-translated region of *CSF1* matched 11 bases at the 5′ end of miR-423-5p, making it a likely target gene. (b) Overexpression or inhibition of miR-423-5p were associated with respective decreases or increases in CSF1 expression (both mRNA and protein) (*p* < 0.05) and luciferase activity (*p* < 0.05). (c) When miR-423-5p expression increased, the number of G0/G1 phase cells and the expression of CDKN1A protein increased while estradiol concentrations in the cell culture solution decreased (*p* < 0.05). However, when miR-423-5p expression decreased, the number of S phase cells increased and E2 concentrations increased while the expression of CDKN1A protein decreased (*p* < 0.05).

**Conclusions:**

Colony stimulating factor 1 is a target gene of miR-423-5p and that it may regulate ovarian response to ovulation induction by affecting granulosa cells proliferation and estrogen secretion.

## Background

Controlled ovarian stimulation (COS) is one of the key steps in in vitro fertilization (IVF), in which exogenous gonadotropins are used to induce the growth and development of multiple follicles during an ovarian cycle. However, due to the different sensitivities of patients to ovulation-promoting drugs, ovarian response significantly varies among different individuals, and even within the same individual between different cycles [1]. In our previous study [2], we found that miR-423-5p expression in the granulosa cells of patients with high ovarian response to exogenous gonadotropins (the patients who had over 14 oocytes retrieved in IVF treatment, hereinafter referred to as “ovarian hyperresponders”) decreased significantly, suggesting that it may play an important role in the regulation of ovarian response. Indeed, MiR-423-5p may alter the expression level of its target genes to cause changes in the number and function of granulosa cells and may increase the sensitivity of ovarian hyperresponders to ovulation-inducing drugs. Some researchers have found that miR-423-5p is important in the development of tumor cells, it can regulate tumor cell proliferation and increase their invasiveness [3, 4]. To the best of our knowledge, no study has reported the role of miR-423-5p in regulating ovarian response.

MicroRNA (miRNA) is an endogenous non-coding small RNA that measures about 21–25 nucleotides. It is involved in post-transcriptional gene regulation by targeting mRNAs for expression, degradation, or translational repression [5, 6]. These miRNAs play key roles in various physiological activities, such as cell proliferation, differentiation, apoptosis, migration, and metabolism. Studies have also shown that miRNA expression is closely related to the regulation of ovarian function: miR-27a-3p, miR-132, miR-133b, miR-212, and miR-224 are involved in regulating ovarian hormone secretion [7–14]; miR-21, miR-15a, miR-105, miR-141-3p, and miR-143 are involved in regulating ovarian cell proliferation and apoptosis [15–24]; and miR-130b, miR-224, miR-378, and miR-383 are involved in regulating follicular growth and egg maturation [25–32]. However, few studies have focused on the link between miRNAs and ovarian response, which is key to ovarian function.

Ovarian granulosa cells play an important role in the regulation of follicular development [33]. There are abundant receptors on the surface of granulosa cells, such as follicle-stimulating hormone (FSH), luteinizing hormone (LH), and estrogen receptors. When FSH binds to granulosa cell surface receptors, it stimulates granulosa cell proliferation and aromatase activity and promotes estradiol (E_2_) synthesis and secretion. Estrogen and FSH synergistically upregulate FSH and LH receptor expressions on granulosa cell surface, further promoting E_2_ synthesis and allowing rapid follicle growth [34, 35]. The granulosa-like tumor cell line, KGN, originated from a 63-year-old Japanese woman with a Stage III granulosa cell carcinoma in 1984 and is often used to study the function of human granulosa cells [36]. Compared with primary cultured granulosa cells, KGN cells have a stable genetic background; furthermore, the experimental results obtained with the latter are unaffected by individual differences and are easy to repeat, making the results more accurate and reliable.

In the present study, we used TargetScan to predict the possible target genes of hsa-miR-423-5p, before seeking to verify the target gene by dual luciferase reporter gene system, quantitative real-time polymerase chain reaction (qRT-PCR), Western blot, and other techniques in KGN cells. In addition, we explored the role and mechanism of hsa-miR-423-5p and its target gene in regulating ovarian response to ovulation induction. We specifically hypothesized that miR-423-5p altered abundance of proteins involved in ovarian granulasa cells proliferation and estrogen secretion.

## Materials and methods

### MicroRNA target gene prediction

The TargetScan software was developed by Lewis et al. [37] and is used to predict the target genes of mammalian miRNAs. It is the first generation of predictive software and uses an algorithm designed according to the basic rules of seed complementation. Target genes are predicted based on the inter-species conservation of miRNA target mRNA sequences. TargetScan introduced the false positive rate for the first time to evaluate the prediction results. The software predicts that a given target gene has a low false positive rate and is widely used in the prediction of miRNA target genes. This study was conducted using the TargetScan database (http://www.targetscan.org/) combined with a literature review to predict the potential target genes for miR-423-5p.

### KGN cell culture and transfection

Granulosa-like tumor cell line KGN cells were donated by Professor Xu Wenming from the Second Hospital of West China, University of Sichuan. All cells were cultured in Dulbecco’s modified Eagle’s medium (DMEM/F12; HyClone, USA) containing 20% fetal bovine serum (Sijiqing, China) and 1% antibiotics (Streptomycin, Penicillin; Gibco, USA). The cells were incubated in a humidified incubator maintained at 37 °C with 5% CO_2_, and the culture medium was replaced every 24 h. After 24 h of cultivation, the cells have grown adherently, most of them were round, and a few were fusiform or polygonal, dark particles can be seen on the cell surface. After 48 h, the cells proliferated significantly, they were uniform in size, spread evenly in a six-well plate, and were fusiform or polygonal. The elongated pseudopods of cells were connected to each other and the particles in the cytoplasm were abundant. Cells were seeded at 1 × 10^5^ cells per well in a six-well plate prepare for transfection when the cells were growing well. Independent experiments were repeated in triplicate.

MicroRNA lentiviral vectors, including hsa-miR-423 expression vector (hereinafter referred to as “lentivirus”) and the control, hsa-miR-423-5p inhibitor (hereinafter referred to as “inhibitor”) and the control, were used to transfect KGN cells. All viral vectors were designed and supplied by the GeneCopoeia company (USA; catalog no. LPP-HmiR0276-MR03, LPP-CmiR0001-MR03, LPP-HmiR-AN0492-AM03, and LPP-CmiR-AN0001-AM03). After removing the old culture solution and the corresponding miRNA lentiviral suspension were separately added to the KGN cells. Gently mix to bring the virus suspension into contact with the cells, and incubate in a 5% CO_2_, 37 °C incubator for 48 h. Cells were harvested for qRT-PCR and Western blot analysis.

### Quantitative real-time polymerase chain reaction (qRT-PCR)

Total RNA was extracted from the KGN cells with Trizol reagent (Sigma, USA), and miR-423-5p expression was measured using an All-in-One™ miRNA qRT-PCR Detection Kit (GeneCopoeia, USA), each following the manufacturer’s protocols. Target gene expression was measured by an All-in-One™ First-Strand cDNA Synthesis Kit (GeneCopoeia, USA) and an All-in-One qPCR Mix (GeneCopoeia, USA). For normalization, U6 (a small nuclear RNA) was used as the endogenous control for hsa-miR-423-5p and GAPDH was used as the endogenous control for the target gene, respectively. The cycle threshold (Ct) was defined as the number of cycles required for the fluorescent signal to cross the threshold in real-time PCR, and ΔCT was calculated by subtracting the Ct values of the internal control from the Ct values of the corresponding gene. Finally, relative expression levels were determined by the 2^−ΔΔCt^ method [38]. The relative expression levels of miRNA and mRNA in each sample were tested in triplicated.

### Western blotting analysis

After the medium was aspirated, the cells were washed twice with phosphate-buffered saline (PBS), before adding the RIPA buffer (Thermo, 89,900, USA) to lyse the cells fully. The lysed cell fluid was added to a 1.5 mL centrifuge tube and centrifuged at 12,000×*g* for 1 min at 4 °C. The protein concentration was determined by a BCA protein assay (Thermo, USA). Protein were separated on 10% sodium dodecyl sulfate–polyacrylamide gel electrophoresis and transferred to a polyvinylidene difluoride membrane. We then prepared 5% skim milk powder as a blocking solution with 1 × PBST (PBS + 0.2% Tween-20) (Sigma, USA). The protein membrane was rinsed, transferred to the blocking solution, and shaken slowly on a shaker at room temperature for 60 min. The blocking solution was aspirated, and the diluted primary antibody (1:1000, Santa Cruz, sc-365,779, USA) was added and incubated overnight at 4 °C. Then the membranes were washed three times and incubated with diluted rabbit anti-mouse IgG-HRP (1:6000, Santa Cruz, sc-358,917, USA) for 1 h. After washing three times with PBST, we detected the protein signal using Clarity Western ECL Substrate (Bio-Rad Laboratories, USA).

### Dual luciferase reporter assay

The 3′ untranslated region (UTR) of the colony stimulating factor 1 (CSF1) mRNA containing the miR-423-5p binding site was cloned into the restriction sites of a CSF1 luciferase reporter vector. This work was done by GeneCopoeia (catalog no. HmiT003149-MT06). KGN cells transfected with miR-423 lentivirus or inhibitor (1 × 10^5^) were seeded into 24-well plates and co-transfected with reporter or control plasmid (provided by GeneCopoeia; catalog no. CmiT000001-MT06). Luciferase assay was assessed using the Luc-Pair™ Duo-Luciferase Assay Kit (GeneCopoeia, USA), following the manufacturer’s instructions. Three wells of cells were used per group.

### Cell-cycle analysis

We harvested control and lentivirus treated KGN cells. EDTA-free trypsin was added to the cells, and the mixture was centrifuged at 750×*g* for 5 min and washed twice with cold PBS. Then, the cells were fixed in ice-cold 70% ethanol overnight at 4 °C. The next day, the cells were centrifuged briefly and washed twice with PBS, before being resuspended in PBS buffer containing RNase A and incubated at 37 °C for 30 min in the dark. The cells were then stained with propidium iodide at room temperature for 30 min, kept in the dark, and processed in a BD LSRFortessa™ flow cytometer (BD Biosciences, USA). About 1 × 10^5^ cells were used to analyze the stage of the cell cycle. Independent experiments were repeated in triplicate.

### Estradiol assay

Cell culture medium (1 × 10^5^ cells) was collected, centrifuged, and the supernatant was extracted. Electro-chemiluminescence immunoassay (ECLIA) was used to measure the E_2_ concentration. ECLIA was performed on Roche Cobas E601 equipment (Roche, Swit). The reagent used in the equipment was Roche’s estradiol detection reagent (Roche, 03000079122, Swit). It contains streptavidin-coated magnetic microparticles (0.72 mg/ml), biotinylated rabbit anti-estradiol antibody (45 ng/l) and Ru (bpy) 32+ labeled estradiol-peptide (2.75 ng/ml). Samples and reagents were loaded in the equipment at relevant positions. The sample volume used for detection of E_2_ by ECLIA was 35 μl. The ECLIA were performed as the manufacturer’s instructions. Once sample is loaded the equipment automatically performed and released the results. For ECLIA calibrators and controls were run as manufacturer’s protocol. The measurement interval was 5.00–4300 pg/ml. The intra and inter coefficients of variation were 1.4–4.9%. The assay was repeated three independent times.

### Statistical analysis

All quantitative data are presented as means ± standard error of the mean, with at least three biological replicates used per analysis. Two-tailed Student’s t-test was utilized to analyze the significance of difference between two groups, whereas categorical data were analyzed using the χ^2^ or Fisher exact tests. *P* < 0.05 was considered to indicate a statistically significant difference. All statistical analyses were performed using PASW Statistics for Windows, Version 18.0 (SPSS, Inc., Chicago, IL, USA).

## Results

### *CSF1* is the predicted target gene of hsa-miR-423-5p

Transfection efficiency exceeded 85% by microscopy 48 h after the KGN cells were transfected with the hsa-miR-423-5p lentivirus. Of note, transfection with the lentivirus or inhibitor caused hsa-miR-423-5p expression to be increased or decreased in KGN cells, respectively (Supplemental Fig. 1a). It was predicted that hsa-miR-423-5p targeted *CSF1*, an important cytokine involved in regulating macrophage proliferation, differentiation, and function. The predicted sequences to which hsa-miR-423-5p binds in the 3′-UTR of *CSF1* are shown in Supplemental Fig. 1b.

### Hsa-miR-423-5p negatively regulates the expression of CSF1

**Fig. 1:**
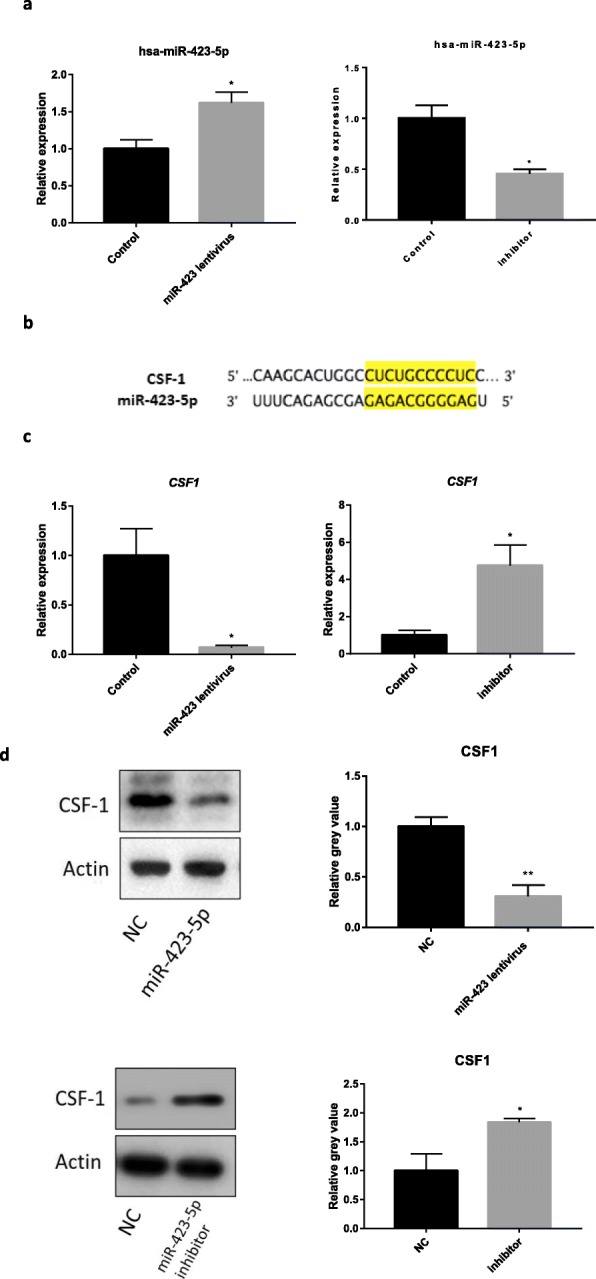
**a** Transfection with the lentivirus or inhibitor caused hsa-miR-423-5p expression to be increased or decreased in KGN cells, respectively. The black bars were controls, and the grey bar was hsa-miR-423 transfection group or hsa-miR-423-5p inhibitor transfection group. (*p < 0.05). **b** The predicted sequences to which hsa-miR-423-5p binds in the 3′-UTR of *CSF1* are shown in yellow shadow. **c** Hsa-miR-423-5p negatively regulates the expression of *CSF1*. Hsa-miR-423-5p overexpression led to reduced *CSF1* expression, whereas the use of inhibitors led to increased *CSF1* expression, in KGN cells. **d** Overexpression of hsa-miR-423-5p reduced the expression of CSF1 protein, and decreased expression of hsa-miR-423-5p increased the expression of CSF1 protein. The bar graphs indicate the mean ± SEM. The black bars were controls, and the grey bar were hsa-miR-423 transfection groups or hsa-miR-423-5p inhibitor transfection groups (*p < 0.05,** *p* < 0.01)

Western blot assays and qRT-PCR further indicated that hsa-miR-423-5p negatively regulates the expression of CSF1, in KGN cells (Fig. 1c, d; *P* < 0.05). Accordingly, when KGN cells were transfected with hsa-miR-423 lentivirus, the expressions of CSF1 mRNA and protein both decreased significantly. By contrast, when KGN cells were transfected with the hsa-miR-423-5p inhibitor, the expressions of CSF1 mRNA and protein increased significantly.

### Dual luciferase reporter gene confirmed that *CSF1* is a target gene of hsa-miR-423-5p

**Fig. 2:**
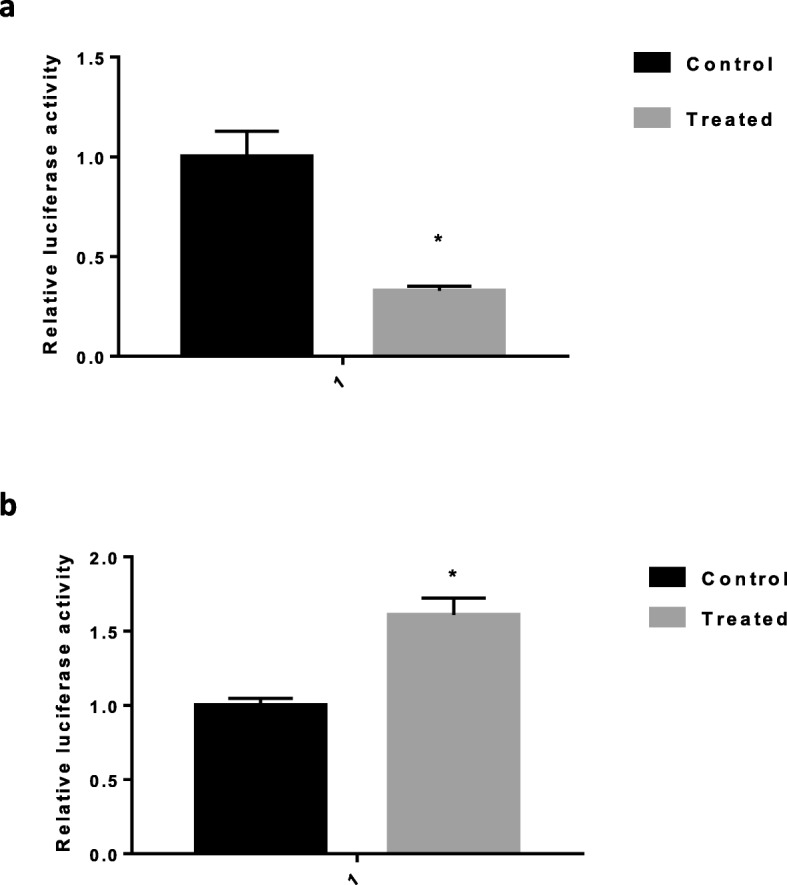
CSF-1 is a target gene of hsa-miR-423-5p. **a** Firefly luciferase activity was significantly decreased in the hsa-miR-423-5p transfected KGN cells compare with the control cells. The black bar was control, and the grey bar was hsa-miR-423 transfection group. **b** Firefly luciferase activity was significantly increased in the hsa-miR-423-5p inhibitor transfected KGN cells compare with the control cells. The black bars was controls, and the grey bar was hsa-miR-423-5p inhibitor transfection group. The data are presented as the mean ± SEM (*p < 0.05)

Next, we examined whether hsa-miR-423-5p could directly regulate *CSF1* expression in the under- or overexpression of hsa-miR-423-5p KGN cells. Our results show that firefly luciferase activity was significantly decreased in the hsa-miR-423-5p overexpression KGN cells (Fig. 2a) and was significantly increased in the hsa-miR-423-5p under-expression KGN cells compared with the control cells (Fig. 2b). These data provide strong evidence that the hsa-miR-423-5p inhibits *CSF1* gene expression by directly binding to sites within the 3′-UTR of *CSF1*.

### Hsa-miR-423-5p could influence cycle proliferation of KGN cells

**Fig. 3:**
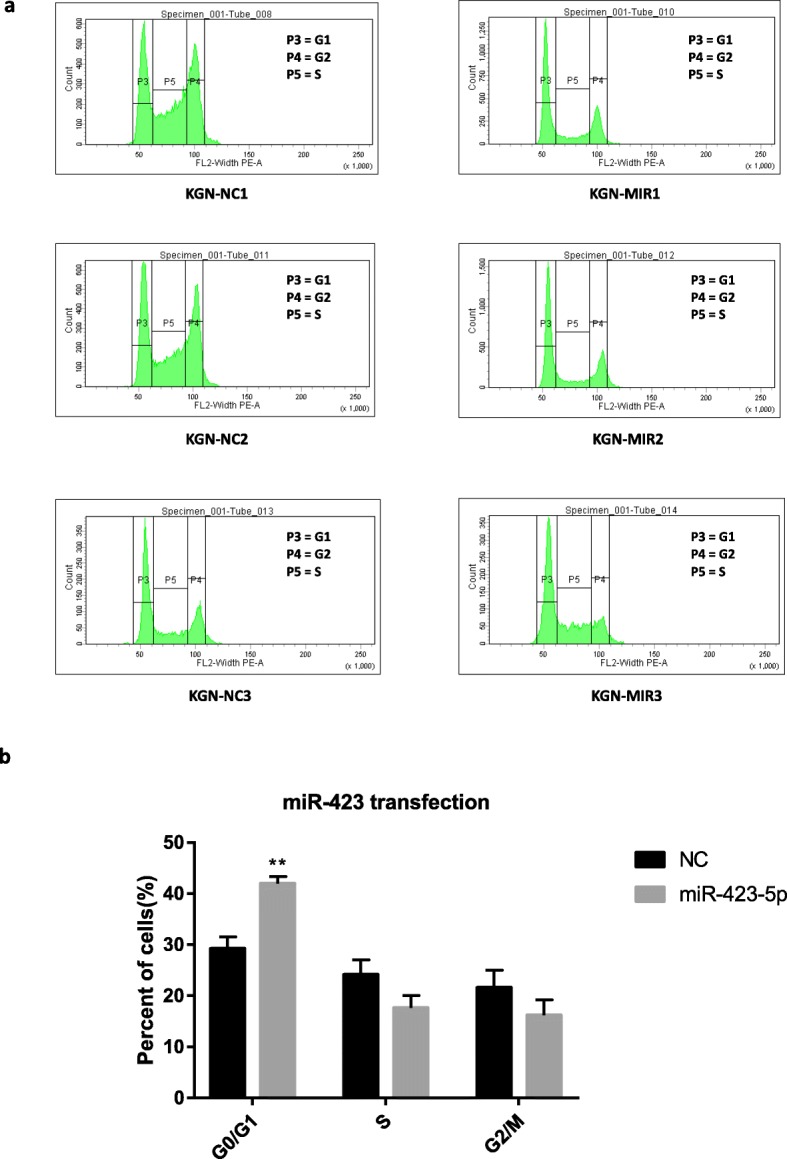
Overexpression of hsa-miR-423-5p blocked the cell cycle progression of KGN cells. **a** Flow cytometric analysis showing the cell cycle distribution (G0/G1, S and G2/M phases) of KGN cells transfected with hsa-miR-423 lentivirus or the negative control (NC). **b** Bar graphs showing the percentages of cells in G0/G1, S and G2/M phases of the cell cycle after transfection with hsa-miR-423 lentivirus. The black bars were controls, and the grey bar were hsa-miR-423 transfection group. The bar graphs indicate the mean ± SEM (**p < 0.01)

**Fig. 4:**
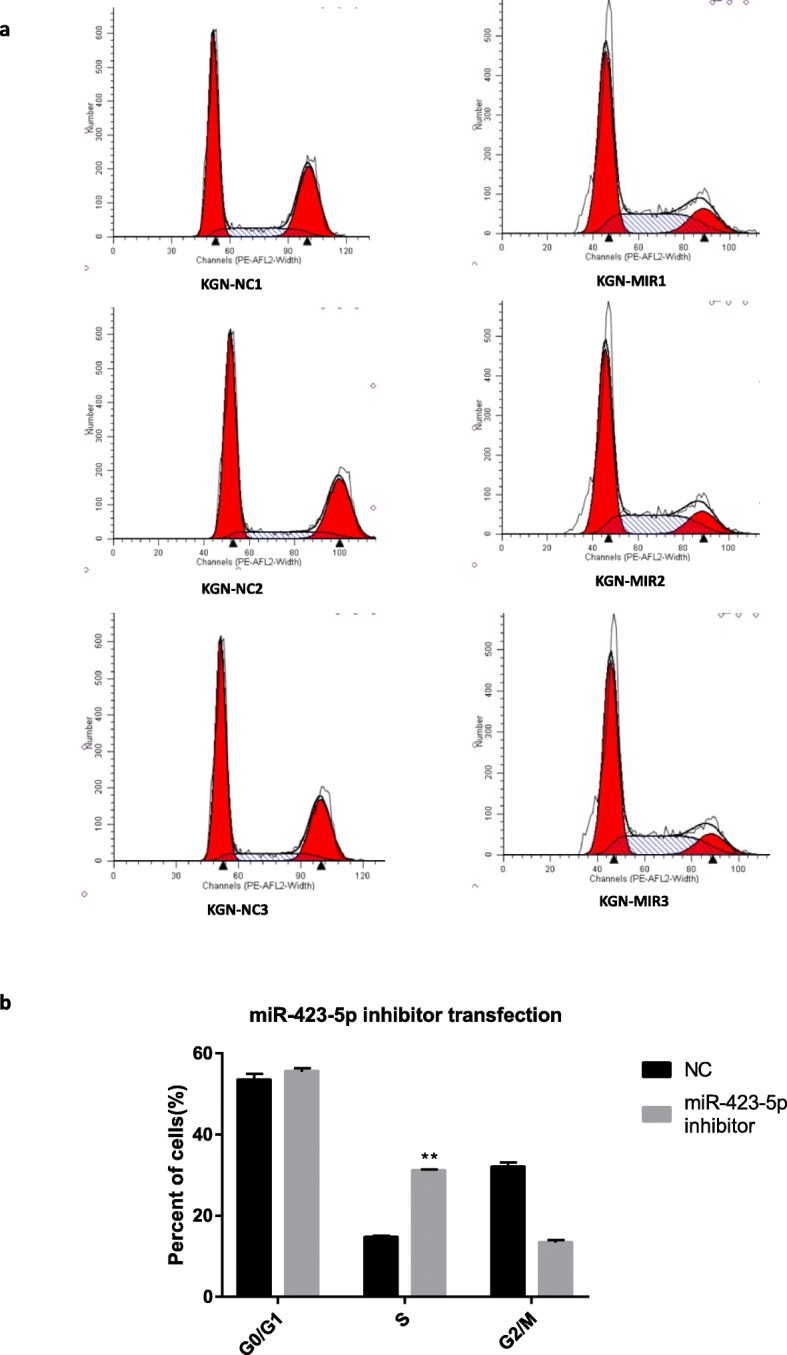
Decreased expression of hsa-miR-423-5p enhanced cell cycle progression of KGN cells. **a** Flow cytometric analysis showing the cell cycle distribution (G0/G1, S and G2/M phases) of KGN cells transfected with hsa-miR-423-5p inhibitor or the negative control (NC). **b** Bar graphs showing the percentages of cells in G0/G1, S and G2/M phases of the cell cycle after transfection with hsa-miR-423-5p inhibitors. The black bars were controls, and the grey bar were hsa-miR-423-5p inhibitor transfection group. The bar graphs indicate the mean ± SEM (**p < 0.01)

To understand the role of hsa-miR-423-5p in modulating the cell cycle of KGN cells, we performed flow cytometric analysis after transfection of the hsa-miR-423-5p lentivirus or inhibitor. Overexpression of hsa-miR-423-5p resulted in a significant increase (*P* < 0.01) in the percentage of cells in the G0/G1 phase compared with the negative control cells (Fig. 3). This result indicated that overexpression of hsa-miR-423-5p could induce KGN cell-cycle arrest, with the inhibition of cell proliferation brought about by impeding transition from the G1 to the S phase of the cell cycle. By contrast, the hsa-miR-423-5p inhibitor caused an increase in the number of cells in the S phase (*P* < 0.01). The results of these investigations are summarized in Fig. 4.

### Hsa-miR-423-5p promotes the expression of CDKN1A

**Fig. 5:**
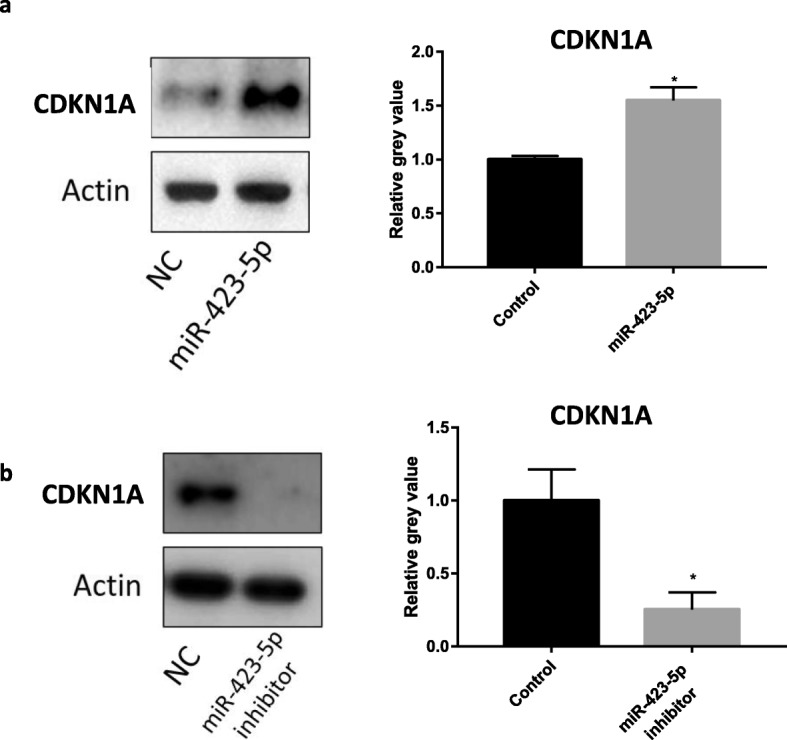
Hsa-miR-423-5p affects the expression of CDKN1A protein. **a** The expression of CDKN1A protein was significantly upregulated by hsa-miR-423-5p overexpression. **b** The expression of CDKN1A protein was downregulated by inhibited expression of hsa-miR-423-5p. The black bars were controls, and the grey bar were hsa-miR-423 transfection groups or hsa-miR-423-5p inhibitor transfection groups. The bar graphs indicate the mean ± SEM. (*p < 0.05)

Cyclin-dependent kinase inhibitor 1A (CDKN1A) is a negative regulator of the cell cycle. The expression of CDKN1A protein was significantly upregulated by hsa-miR-423-5p overexpression, while its expression was downregulated by inhibited expression of hsa-miR-423-5p (Fig. 5).

### Hsa-miR-423-5p affects the secretion of E_2_ in KGN cells

**Fig. 6:**
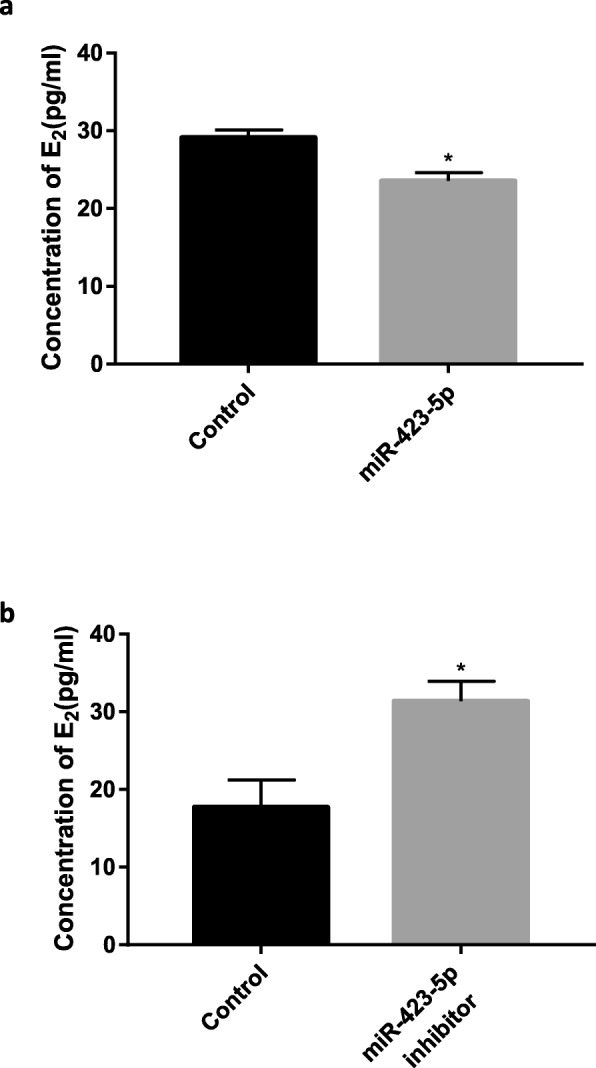
Hsa-miR-423-5p affects the secretion of E_2_ in KGN cells. **a** The concentration of E_2_ in cell culture medium decreased significantly after hsa-miR-423-5p overexpression. **b** The concentration of E_2_ in cell culture medium increased significantly after hsa-miR-423-5p expression decreased. The black bars were controls, and the grey bar was hsa-miR-423 transfection group or hsa-miR-423-5p inhibitor transfection group. The bar graphs indicate the mean ± SEM. (*p < 0.05)

The concentration of E_2_ in the KGN cell culture medium, as measured by ECLIA, decreased significantly (*P* < 0.05) after hsa-miR-423-5p overexpression (Fig. 6a). By contrast, the hsa-miR-423-5p inhibitor caused an increase in E_2_ levels in the KGN cell culture medium (Fig. 6b). These results indicated that hsa-miR-423-5p could affect the secretion of E_2_ from KGN cells.

## Discussion

In the present study, we found that *CSF1* is a target gene of hsa-miR-423-5p. Moreover, we demonstrated that hsa-miRNA-423-5p could influence cycle proliferation of KGN cells, and this effect may be achieved by affecting the expression of CDKN1A. Through measuring the E_2_ concentration of cell culture medium, we show that the secretion of E2 in KGN cells affected by hsa-miR-423-5p. These results suggest that the altered expression of hsa-miR-423-5p and its target gene *CSF1* in ovarian granulosa cells maybe the reason of high ovarian response to exogenous gonadotropins.

Previous studies have shown that *CSF1* is a target gene for a variety of miRNAs. For example, miR-1207-5p targeting *CSF1* inhibits the implantation and metastasis of lung cancer [39]; miR-148b targets *CSF1* and other genes that inhibit the development of breast cancer [40]; miR-142-3p targets *CSF1* to induce the conversion of monocytes to macrophages [41]; and miR-214 targets *CSF1* to regulate the proliferation, invasion, and migration of gastric cancer cells [42]. To date, there have been no reports of miR-423-5p targeting *CSF1*, as we have shown in this study. We confirmed that *CSF1* is a target gene of hsa-miR-423-5p for the first time.

Cytokines are small proteins that bind to receptors on the surfaces of cell membranes. They are involved in promoting cell growth and in regulating immune responses, as well as having a role in inflammatory reactions. Studies have shown that cytokines play important roles in follicular growth and embryonic development. Ovarian granulosa cells secrete a variety of cytokines to promote follicular growth, ovulation, hormone synthesis, and secretion [43–45]. Cytokine expression levels are also closely related to the maintenance of normal follicular development and to steroid hormone secretion in the ovary [46]. Colony stimulating factor 1 is an important cytokine that mainly acts on mononuclear macrophage cell lines, where it is involved in regulating their proliferation, differentiation, and function [47]. Previous studies have shown that CSF1 is also key to the regulation of female reproductive function. In this study, we showed that *CSF1* was a target gene of miR-423-5p and that CSF1 most likely regulated ovarian reactivity to control ovarian stimulation by affecting the cell cycle and hormone secretion.

Colony stimulating factor 1 secreted by ovarian granulosa cells through autocrine or paracrine routes. Once released, it binds to cell surface receptors and affects intracellular metabolism, oocyte meiosis, and follicular growth and maturation. As early as 1995, Nishimura et al. [48] first reported that CSF1 affected follicular development and ovulation in rats. A significant increase in the ovulation rate was observed in female rats after CSF1 treatment. Later Araki et al. [49] found that the number of ovulations, antral follicles, and mature follicles were lower in osteopetrotic (op/op) mutant mice when compared with normal litters. The op/op mice lack the coding region for the *CSF1* gene and are completely devoid of CSF1. Moreover, the number of granulosa cells and the proliferative capacity of antral follicles were also reduced in the op/op mice. When the researchers injected supplementary CSF1 into the op/op mice, the numbers of antral follicles, mature follicles, and granulosa cells around the follicle increased. Therefore, they concluded that CSF1 promoted ovarian granulosa cell proliferation and that it participated in regulating follicular production and ovulation. Cohen et al. [50] also observed a decrease in the ovulation rate of op/op mice. In 1997, researchers showed that CSF1 and its mRNA were expressed in human follicular fluid, and confirmed for the first time that CSF1 was involved in regulating human follicular development [51]. Soon after, Nishimura et al. [52] observed that the concentration of CSF1 in the serum increased gradually with the duration of COS, peaking between the egg retrieval day and 2 days later. Moreover, the concentration of CSF1 in the follicular fluid was significantly higher than that in the serum on the day of egg retrieval, and the concentration of CSF1 in the follicular fluid containing the ovum was higher than in that of the empty follicle. This study suggested that CSF1 may be involved in regulating egg maturation and ovulation. In other research, it was reported that the concentration of CSF1 in the follicular fluid was significantly higher than that in the serum. Furthermore, the expressions of CSF1 and its receptor were detected in isolated and cultured human luteinized granulosa cells, confirming that CSF1 is a key factor in follicular development [53].

The concentration of CSF1 in serum or follicular fluid is associated with ovarian response. Salmassi et al. [54] observed that there were difference in serum CSF1 levels depending on ovarian response during COS. They found that the higher a ovarian response with greater egg production was associated with a higher serum CSF1 concentration. Lei Huo [55] obtained similar results in patients undergoing IVF treatment, showing that the CSF1 concentration in the follicular fluid on the day human chorionic gonadotropin was given had a positive correlation with the number of eggs obtained. Thus, it is believed that CSF1 can affect ovarian reactivity and is related to egg maturation.

In our previous study [2], bioinformatics analysis of miRNAs with altered expression in ovarian hyperresponders revealed that the target genes of differential miRNAs were enriched in pathways such as cell-cycle regulation. In this study, we found that increased hsa-miR-423-5p can cause a large number of KGN cells to arrest in the G0/G1 phase, with the number of cells in S phase increased when hsa-miR-423-5p expression was inhibited. Although the percentage of S phase cells was no statistical difference between miR-423-5p overexpression group and the negative control, it showed a downward tendency in the miR-423-5p overexpression group compared to the control. This result indicates that the expression level of hsa-miR-423-5p in KGN cells does affect the cell cycle, which is consistent with our previous study and confirms that hsa-miR-423-5p is involved in the proliferation of KGN cells.

To further clarify the mechanism through which hsa-miR-423-5p regulated the cell cycle, we examined the expression of CDKN1A protein. Cyclin-dependent kinase inhibitor 1A is a negative regulator of the cell cycle that inhibits the activity of Cyclin-dependent kinase (CDK) and blocks cells in the G1 phase, thereby inhibiting cell proliferation [56]. When the hsa-miR-423-5p expression was increased, CDKN1A protein expression was upregulated. Conversely, when hsa-miR-423-5p expression was decreased, CDKN1A protein expression was downregulated. Thus, it appears that hsa-miR-423-5p may affect CDK expression and induce cell arrest in the G0/G1 phase, thereby affecting cell proliferation. In patients with high ovarian response to COS, the decreased expression of hsa-miR-423-5p may lead to decreased inhibition of *CSF1* and increased number of S phase cells. In turn, this could result in an abnormal high level of CSF1 in granulosa cells, causing the excessive proliferation of granulosa cells and the development of multiple follicles. This may explain cases of high ovarian response to COS. However, compared with the control group, the percentage of G0/G1 phase cells in miR-423-5p inhibitor group was not significantly reduced. According to our analysis, the possible reason is that the sample size is too small to obtain statistically significant differences in both the S and G0/G1 phases. More samples are required to fully profile the cell proliferation differences between the cells with different miR-423-5p expression in the future.

It is known that high levels of E_2_ are usually associated with high ovarian response in patients [57]. In this study, the E_2_ concentration was measured in the culture medium of KGN cells: concentrations decreased when hsa-miR-423-5p expression increased and concentrations increased when intracellular hsa-miR-423-5p expression decreased. Study have shown that CSF1 can help FSH by promoting E_2_ secretion from ovarian granulosa cells and upregulating FSH receptor expression [58]. Therefore, we hypothesized that hsa-miR-423-5p overexpression inhibited *CSF1* expression and led to a decrease in E_2_ secretion by KGN cells. This effect on hormone secretion was attenuated after hsa-miR-423-5p was inhibited. Downregulation of hsa-miR-423-5p in patients with high ovarian response to COS may lead to an increase in the CSF1 concentration in granulosa cells, thereby stimulating excessive secretion of E_2_ by cells.

Based on these findings, we can state that hsa-miR-423-5p may regulate the proliferation of ovarian granulosa cells and the secretion of E_2_ within a proper range. It appears to do so by negatively regulating the expression of its target gene, *CSF1*, in ovarian granulosa cells. This is characterized by a normal ovarian response. In patients with a high ovarian response, however, hsa-miR-423-5p expression appears to be downregulated, such that the inhibition of *CSF1* is weakened. In turn, this may lead to excessive CSF1 secretion, excessive granulosa cell proliferation, abnormal sensitivity to exogenous gonadotropins, simultaneous development of a large number of follicles being promoted, and abnormal E_2_ elevations. Together, these result in a high ovarian response.

To the best of our knowledge, this is the first study to have linked the expression of granulosa cell miRNAs to the secretion of CSF1. We found an upstream regulatory factor that causes changes in CSF1 concentrations in granulosa cells, thereby adding to current knowledge of the factors associated with ovarian reactivity. However, in this study, we only conducted a preliminary exploration of the mechanism through which miR-423-5p and its target gene, *CSF1*, regulate ovarian response. Further research is therefore needed to identify the signal pathway through which the miR-423-5p targeting of CSF1 regulates ovarian response. In addition, we will try to find suitable targeted inhibitors affecting miR-423-5p/CSF1 pathway in vitro and in vivo to avoid high ovarian response as much as possible. If the research is successful, it may help prevent ovarian hyperstimulation syndrome (OHSS).

## Conclusion

In summary, we present the novel finding that *CSF1* is a target gene of hsa-miR-423-5p. Our results obtained on KGN cells suggest that hsa-miR-423-5p downregulation in the ovarian granulosa cells of patients with high ovarian response abnormally increases CSF1 expression. This may cause a massive proliferation of granulosa cells and an excessive secretion of E_2_, which produce the high ovarian response. These findings are important for further studies on the mechanism of miRNA action in the human ovary.

## Supplementary information

**Additional file 1.**

## Data Availability

The datasets used and/or analysed during the current study are available from the corresponding author on reasonable request.

## Abbreviations

COS: Controlled ovarian stimulation
IVF: In vitro fertilization
miRNA: MicroRNA
FSH: Follicle-stimulating hormone
LH: Luteinizing hormone
E2: Estradiol
qRT-PCR: Quantitative real-time polymerase chain reaction
ECLIA: Electro-chemiluminescence immunoassay
CSF1: Colony stimulating factor 1
CDKN1A: Cyclin-dependent kinase inhibitor 1A
CDK: Cyclin-dependent kinase
OHSS: Ovarian Hyper-stimulation Syndrome
