# Supplementary figures and images for: MiR-423-5p may regulate ovarian response to ovulation induction via CSF1
Source: Reprod Biol Endocrinol. 2020 Apr 7;18:26. doi: 10.1186/s12958-020-00585-0 (PMC7137414; doi:10.1186/s12958-020-00585-0)

**Here are the pictures of** **KGN cells before and after transfection (see blow).**


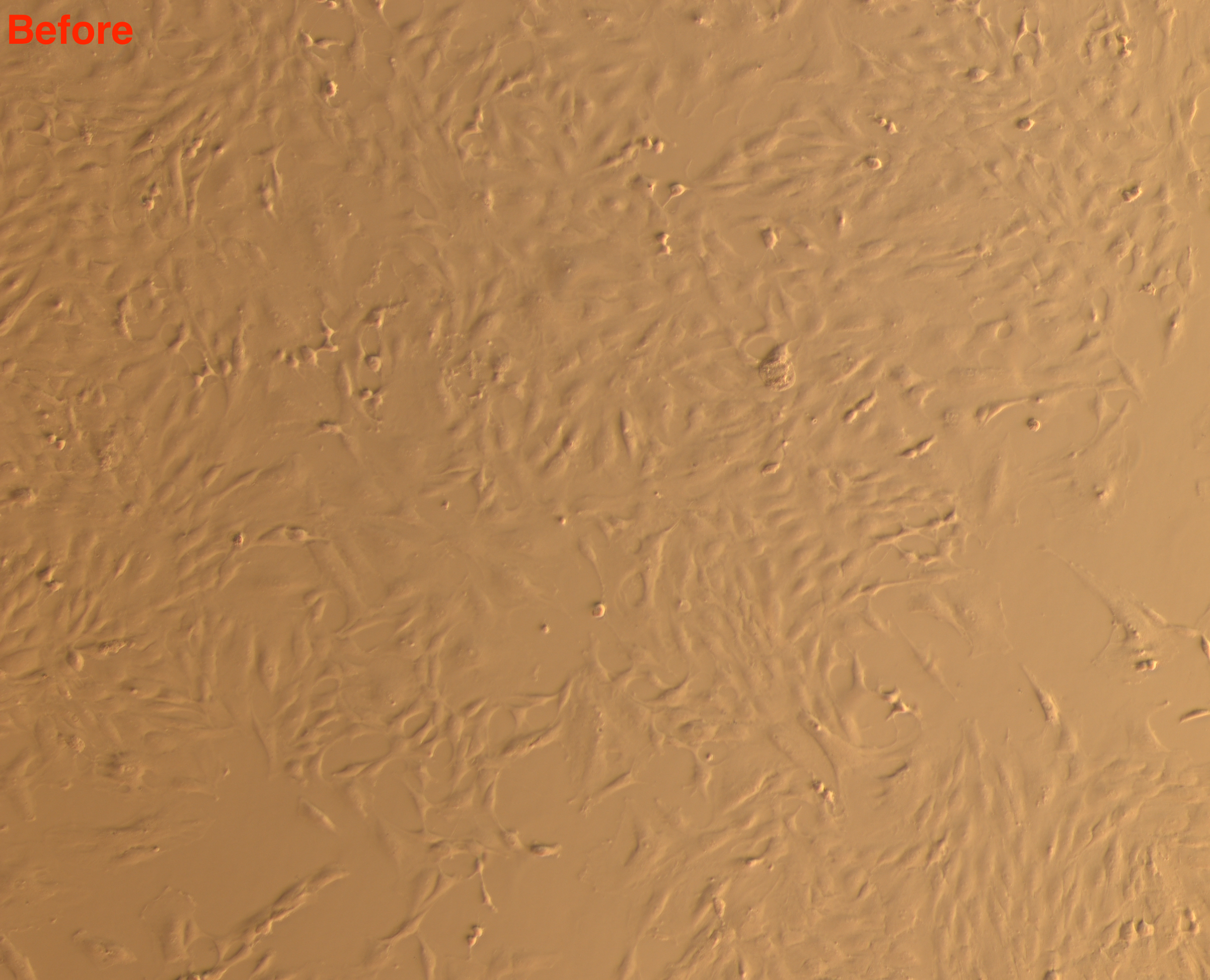

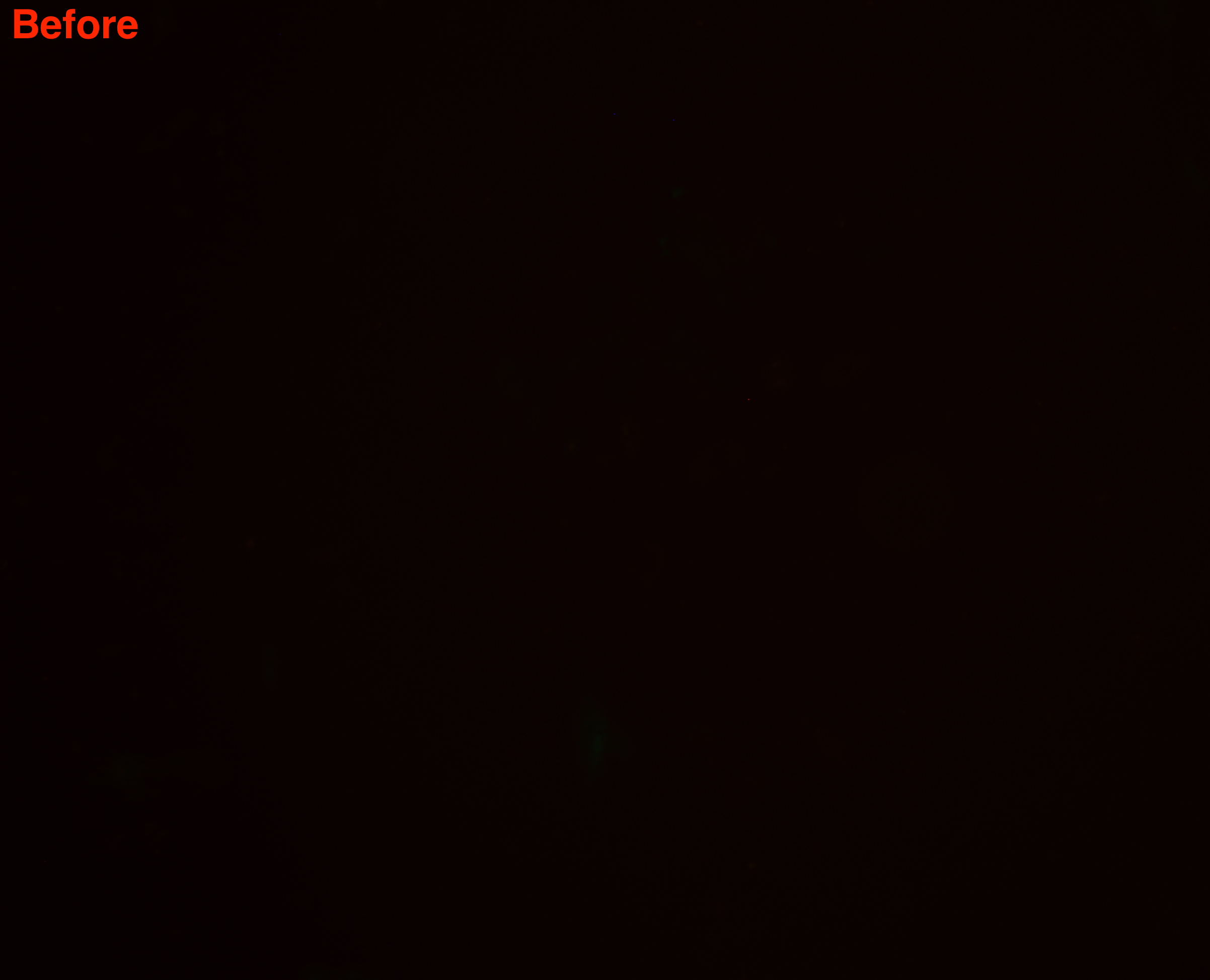


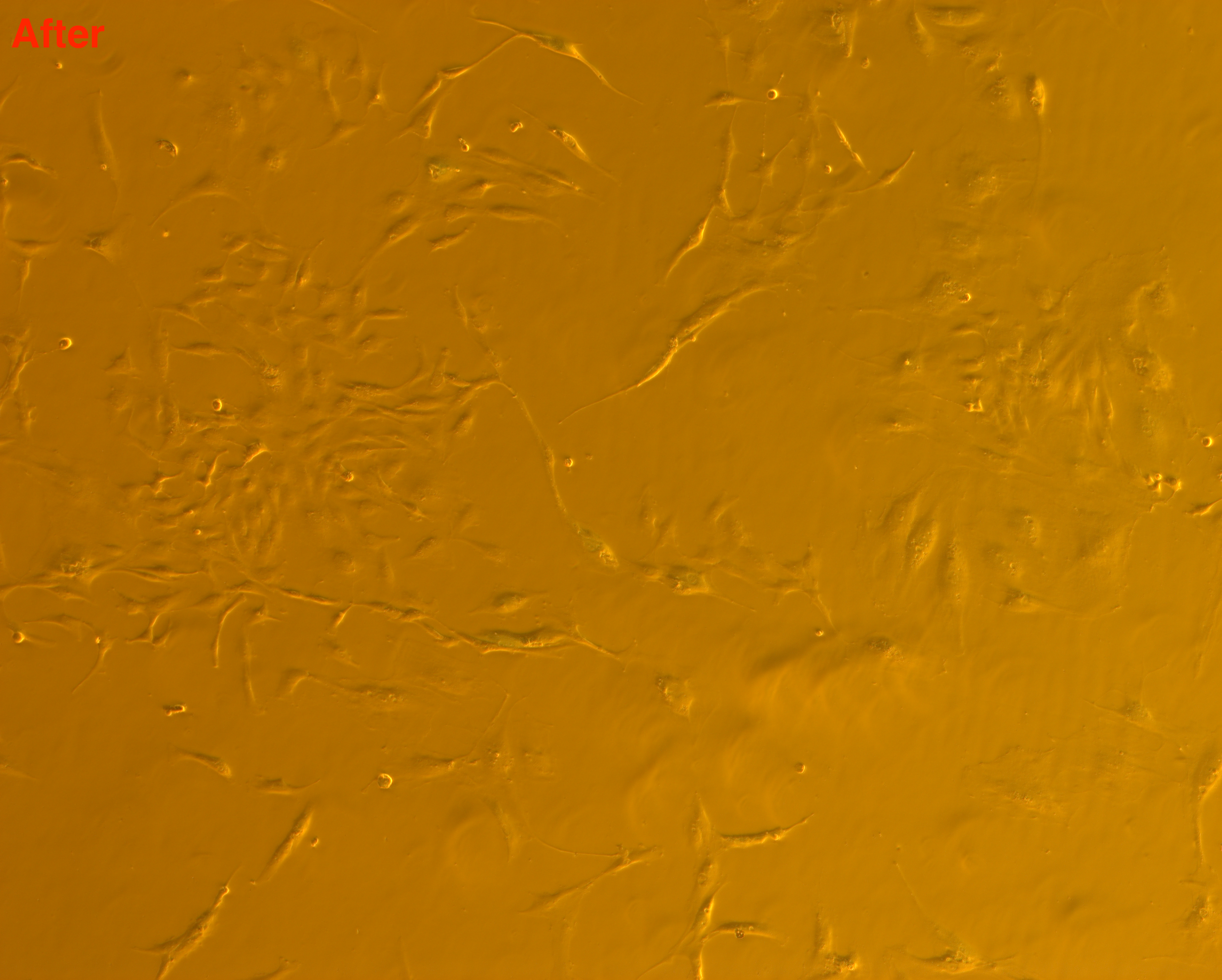


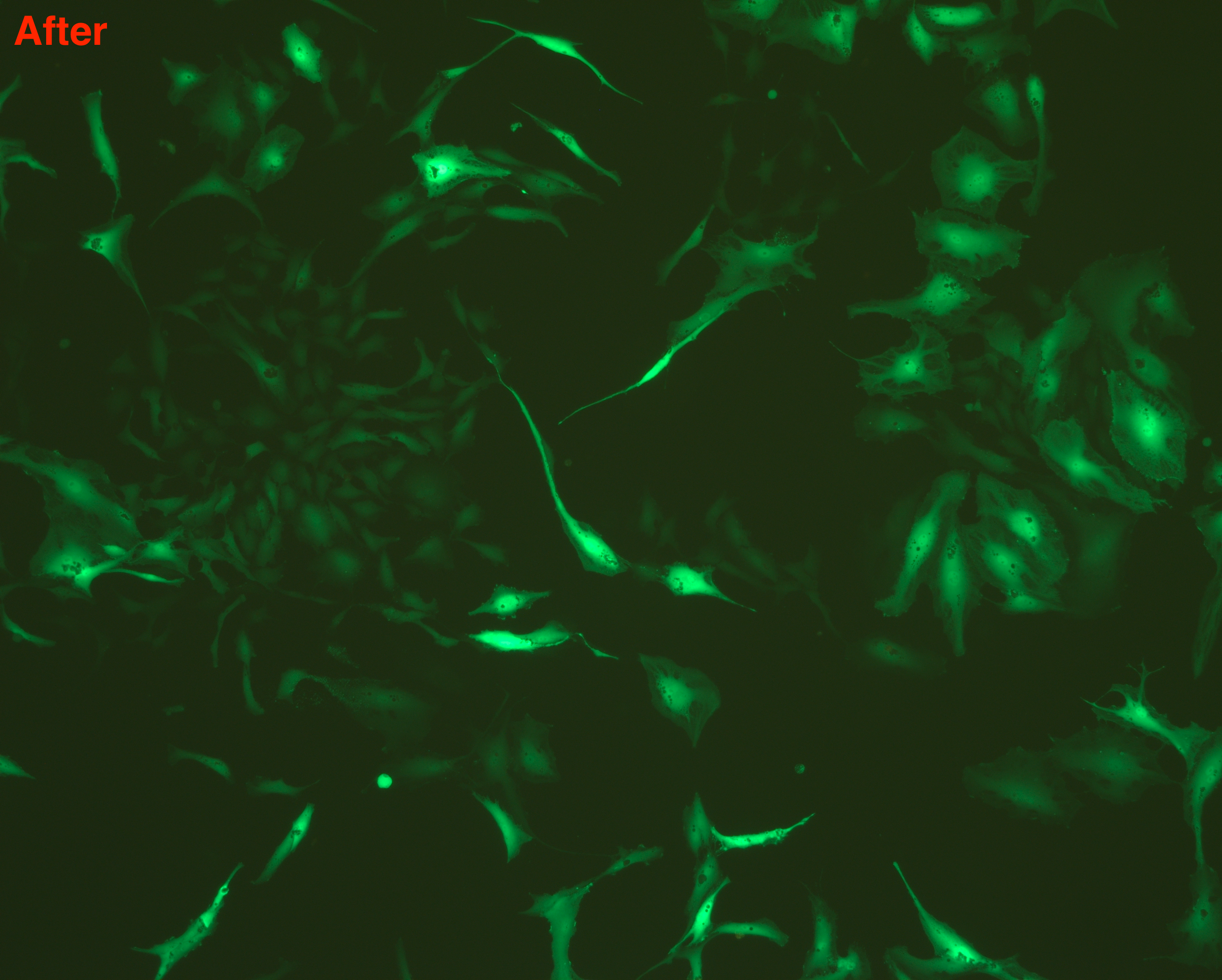

Supplement: Supplementary file 1 — Additional file 1. [file 12958_2020_585_MOESM1_ESM.docx]
